# Association of meal timing with body composition and cardiometabolic risk factors in young adults

**DOI:** 10.1007/s00394-023-03141-9

**Published:** 2023-04-26

**Authors:** Manuel Dote-Montero, Francisco M. Acosta, Guillermo Sanchez-Delgado, Elisa Merchan-Ramirez, Francisco J. Amaro-Gahete, Idoia Labayen, Jonatan R. Ruiz

**Affiliations:** 1 Department of Physical Education and Sports, Faculty of Sports Science, Sport and Health University Research Institute (iMUDS), University of Granada, Carretera de Alfacar s/n, 18071 Granada, Spain; 2 Turku PET Centre, University of Turku, Turku, Finland; 3 Turku PET Centre, Turku University Hospital, Turku, Finland; 4 InFLAMES Research Flagship Center, University of Turku, Turku, Finland; 5 Division of Endocrinology, Department of Medicine, Centre de Recherche du Centre Hospitalier Universitaire de Sherbrooke, Université de Sherbrooke, Sherbrooke, QC Canada; 6 CIBER de Fisiopatología de la Obesidad y Nutrición (CIBEROBN), Instituto de Salud Carlos III, Granada, Spain; 7 Institute for Sustainability and Food Chain Innovation (ISFOOD), University of Navarra, Pamplona, Spain; 8 Navarra Institute for Health Research, IdiSNA, Pamplona, Spain; 9 Department of Health Sciences, Public University of Navarra, Campus de Arrosadia, Pamplona, Spain; 10 Instituto de Investigación Biosanitaria, Ibs.Granada, Granada, Spain

**Keywords:** Chrononutrition, Circadian rhythms, Timing of food intake, Intermittent fasting, Fat mass, Insulin resistance

## Abstract

**Purpose:**

To investigate the association of meal timing with body composition and cardiometabolic risk factors in young adults.

**Methods:**

In this cross-sectional study participated 118 young adults (82 women; 22 ± 2 years old; BMI: 25.1 ± 4.6 kg/m^2^). Meal timing was determined via three non-consecutive 24-h dietary recalls. Sleep outcomes were objectively assessed using accelerometry. The eating window (time between first and last caloric intake), caloric midpoint (local time at which ≥ 50% of daily calories are consumed), eating jetlag (variability of the eating midpoint between non-working and working days), time from the midsleep point to first food intake, and time from last food intake to midsleep point were calculated. Body composition was determined by DXA. Blood pressure and fasting cardiometabolic risk factors (i.e., triglycerides, total cholesterol, high-density lipoprotein-cholesterol, low-density lipoprotein-cholesterol, and insulin resistance) were measured.

**Results:**

Meal timing was not associated with body composition (*p* > 0.05). The eating window was negatively related to HOMA-IR and cardiometabolic risk score in men (*R*^2^ = 0.348, *β* = − 0.605; *R*^2^ = 0.234, *β* = − 0.508; all *p* ≤ 0.003). The time from midsleep point to first food intake was positively related to HOMA-IR and cardiometabolic risk score in men (*R*^2^ = 0.212, *β* = 0.485; *R*^2^ = 0.228, *β* = 0.502; all *p* = 0.003). These associations remained after adjusting for confounders and multiplicity (all *p* ≤ 0.011).

**Conclusions:**

Meal timing seems unrelated to body composition in young adults. However, a longer daily eating window and a shorter time from midsleep point to first food intake (i.e., earlier first food intake in a 24 h cycle) are associated with better cardiometabolic health in young men.

**Clinical trial registration:**

NCT02365129 (https://www.clinicaltrials.gov/ct2/show/NCT02365129?term=ACTIBATE&draw=2&rank=1).

**Supplementary Information:**

The online version contains supplementary material available at 10.1007/s00394-023-03141-9.

## Introduction

The obesity epidemic is one of the leading contributors to the global burden of cardiometabolic diseases and disability, resulting in a significant economic impact on health care systems [1, 2]. However, despite the known consequences of excess body weight, the prevalence of obesity continues to rise, and currently there are more than 1.9 billion adults with overweight or obesity worldwide [1]. Body weight regulation and obesity are highly influenced by several factors such as genetics, physiology, and socioeconomic factors [3]. For instance, in recent years, emerging evidence has shown that the timing of food intake may be a relevant risk factor for obesity and cardiometabolic diseases [4, 5].

The importance of when we eat is tied to our circadian system, which temporally orchestrates numerous metabolic processes across the body over a 24 h period [6, 7]. The circadian system is constituted by a central clock located in the suprachiasmatic nuclei of the hypothalamus and a series of peripheral clocks placed in virtually all cells and tissues of the body [6, 7]. The central clock is primarily controlled by external light via direct connection to the retina, whereas peripheral clocks are regulated by the central clock and external time cues, such as eating, physical activity and sleep among other behavioural tasks [6, 7]. Since different stimuli impact the central and peripheral clocks, the two clock systems become misaligned whenever their respective time cues are out of synchrony [6, 7]. The modern lifestyle promotes this circadian misalignment by allowing exposure to artificial light at night and by allowing access to food all day long [6–8].

Epidemiological data have shown that eating late in the day is associated with greater energy intake, adiposity and worst cardiometabolic health [9–16]. Furthermore, considerable variability in meal timing between non-working days and working days, coined eating jet lag, has been linked to higher body mass index (BMI) [17]. Clinical trials have also revealed that reducing the daily eating window (i.e., the period of time between the first and the last caloric intake) and/or shifting dietary energy intake earlier in the day results in body weight loss or optimized cardiometabolic health in adults with overweight or obesity [9, 18–22]. Nonetheless, one of the main limitations of meal timing studies is the use of clock time to illustrate the timing of food intake, which fails to correctly characterize meal timing in relation to the internal circadian time [10, 23]. Because obtaining measures of dim-light melatonin is not practical in large clinical trials, it has been proposed that the timing of food intake should be assessed in relation to the sleep/wake cycle as a proxy of circadian time [10, 23, 24]. Only a few studies have followed this methodology, indicating that a longer time period from dinner to midsleep point or bedtime is associated with lower adiposity and BMI, respectively [10, 23]. However, these studies also present important limitations, such as the self-reported assessment of sleep timing, solely including anthropometric variables or fat mass measured by non-state-of-the-art methods and the absence of various cardiometabolic risk. Moreover, although sex is known to influence several aspects of biology, physiology and psychology previous studies have not investigated if there are sex differences in the relationship between meal timing with body composition and cardiometabolic risk factors.

Our study was aimed at elucidating the association of meal timing [i.e., eating window, caloric midpoint (the time at which ≥ 50% of daily calories are consumed), eating jet lag, time from midsleep point to first food intake, and time from last food intake to midsleep point] with anthropometry [i.e., body weight, BMI and waist circumference], body composition [i.e., fat mass, lean mass, and visceral adipose tissue (VAT) mass] and cardiometabolic risk factors [i.e., blood pressure, triglycerides, total cholesterol, high-density lipoprotein-cholesterol (HDL-C), low-density lipoprotein-cholesterol (LDL-C), insulin resistance] in young adults. Special attention was paid to the influence of sex on these associations. We hypothesized that a shorter daily eating window, earlier food intake schedule and less variability in meal timing between non-working days and working days would be associated with healthier body composition and cardiometabolic risk factors.

## Materials/subjects and methods

### Participants

A total of 118 young adults (*n* = 82 women; see Flow Chart, Figure S1) took part in this cross-sectional study which includes the baseline measurements of the ACTIBATE study (ClinicalTrials.gov NCT02365129) [25] and follows the STROBE-nut guidelines (Table S1) [26]. All assessments were performed in Granada (Spain) during September-December in 2015 and 2016. The inclusion/exclusion criteria included the following: (i) being 18–25 years, (ii) having a BMI ranging from 18 to 35 kg/m^2^, (iii) being non-smokers, (iv) not being enrolled in a weight loss program, (iii) having a stable body weight (body weight changes < 3 kg over 3 months), (iv) not being physically active (< 20 min on < 3 days/week), (v) not taking any medication or drugs, and (vi) not suffering from any acute or chronic illness.

### Outcome measurements

#### Anthropometry and body composition

Body weight and height were determined with participants wearing light clothing and without shoes using a SECA scale and stadiometer (model 799; Electronic Column Scale, Seca GmbH, Hamburg, Germany). The BMI was calculated as weight (kilograms) divided by height squared (square meters). Waist circumference (centimetres) was assessed midway between the lowest rib and the top of the iliac crest. Alternatively, when participants showed abdominal obesity, waist circumference was determined in a horizontal plane above the umbilicus after exhalation.

Body composition was measured by dual-energy X-ray absorptiometry (DiscoveryWi; Hologic, Inc., Marlborough, MA, USA) strictly following the manufacturer’s instructions, obtaining fat mass, lean mass, and VAT mass. The lean mass index was calculated as lean mass (kilograms) divided by height squared (square meters).

#### Cardiometabolic profile

The systolic and diastolic blood pressure were measured on three different days with an automatic monitor (Omron Healthcare Europe B.V. Hoofddorp, The Netherlands), and the average was used in later analyses. We calculated the mean blood pressure as diastolic blood pressure + 1/3(systolic blood pressure − diastolic blood pressure). Blood samples were collected from the antecubital area in the morning after fasting for > 10 h. All samples were centrifuged, and aliquots of serum were stored at − 80 °C until analysis. All participants were requested to abstain from drugs and caffeine and to avoid moderate-intensity physical activity and vigorous-intensity activity for 24 and 48 h before testing, respectively. Serum glucose, total cholesterol, HDL-C and triglycerides were assessed following standard methods using an AU5832 automated analyzer (Beckman Coulter Inc., Brea CA, USA). LDL-C was then estimated [27]. Serum insulin was measured using the Access Ultrasensitive Insulin Chemiluminescent Immunoassay Kit (Beckman Coulter Inc., Brea, CA, USA). The homeostasis model assessment of insulin resistance (HOMA-IR) was calculated.

Cardiometabolic risk scores were calculated for each sex based on variables included in the diagnosis of Metabolic Syndrome [28]: waist circumference, blood pressure, plasma glucose, HDL-C, and triglyceride concentrations. A *Z*-score was calculated for each variable. The HDL-C standardized values were multiplied by − 1 to be directly proportional to the cardiometabolic risk. The final score was determined as the average of the 5 *Z*-scores. Thus, the cardiometabolic risk score is a continuous variable with a mean of 0 and a standard deviation of 1; higher scores indicating higher risk.

#### Sleep parameters, sedentary time and physical activity assessment

Participants were instructed to wear a triaxial accelerometer (ActiGraph GT3X+, Pensacola, FL, USA) on the non-dominant wrist 24 h a day for seven consecutive days, removing them only when swimming or bathing, and to record information on sleep onset and wakeup times each day in a sleep diary. The midsleep point and other sleep-related outcomes were objectively assessed using an algorithm guided by the participants' reported times. The raw data were processed in R [version 3.1.2, https://www.R-project.org/] using the GGIR package [version 1.5-12, https://cran.r-project.org/web/packages/GGIR/]. Actigraphy recordings were used to determine: (i) sleep onset (time at which the subject fell asleep); (ii) sleep offset (time at which the subject woke up); and (iii) sleep duration (time between falling asleep and waking up). Daytime naps were not considered. Those participants registering less than 16 h/day of wear time for more than 4 days and/or not having data from at least one weekend day were excluded from the final analysis. We used these sleep variables to calculate the midsleep point (the middle time point between sleep onset and sleep offset) as follows: midsleep point (local time) = (sleep duration/2) + sleep onset [24]. We determined the midsleep point for non-working days and working days separately. Subsequently, the midsleep point for the week was calculated as a weighted mean, which was used in later analyses. Last, we computed social jetlag (a proxy of the discrepancy between social and biological time) by subtracting each participant’s midsleep point for working days from non-working days [24].

We estimated the time spent in sedentary behavior and different physical activity intensities (i.e., light, moderate, vigorous, and moderate to vigorous) using age-specific cut points [29].

#### Dietary assessment and meal timing

Dietary intake and meal timing were recorded using three non-consecutive 24 h dietary recalls (one of them for a non-working day) distributed over three weeks with the participants unaware of when their diets were going to be recorded. In face-to-face interviews performed by trained dietitians, participants were asked to recall all food consumed on the previous day, using photographs of portion sizes [30], as well as the time of the meal event. Data recorded in the interviews were independently introduced by two dieticians in the EvalFINUT^®^ software When the coefficient of variance between the two datasets was > 5%, a third dietician reintroduced the data obtained from the 24-h recalls in the EvalFINUT^®^ software and the mean of the two datasets with the best agreement (i.e., lower coefficient of variance) was used. From these data, we calculated the following meal timing variables:

The eating window (i.e., the period of time between the first and the last caloric intake) was calculated for each day [31]. The caloric midpoint is defined as the time at which ≥ 50% of daily calories are consumed and is expressed in local time [12]. We determined the eating window and the caloric midpoint for non-working days and working days. Subsequently, the value for the week was calculated as a weighted mean, which was used in later analyses. Eating jetlag (i.e., the variability of the eating midpoint between non-working days and working days) was determined as [24]: eating midpoint for non-working days − eating midpoint for working days. The time from the midsleep point to first food intake and the time from last food intake to the midsleep point were also calculated.

Dietary patterns and quality were determined by analysing the participants' data from the 24 h recalls and a validated food frequency questionnaire [32]. Three The following Mediterranean dietary patterns [33–35] were computed: the a priori Mediterranean dietary pattern [33], the Mediterranean diet score, and the dietary quality index for a Mediterranean diet [34, 35]. Adherence to the Dietary Approaches to Stop Hypertension guidelines was also calculated. The diet quality index [36] and the dietary inflammatory index [37], were also determined. Breakfast consumers were defined as consuming breakfast in all of the three non-consecutive 24 h dietary recalls, whereas breakfast skippers were defined as not consuming breakfast on at least one of the three non-consecutive 24 h dietary recalls.

### Statistical analyses

The distribution of the variables was verified using the Shapiro–Wilk test, skewness and kurtosis values, visual checking of histograms, and Q–Q and box plots. The descriptive parameters are reported as mean and standard deviation when normally distributed, or medians (interquartile range) when a Gaussian distribution was not found. Triglycerides, total cholesterol, HDL-C, LDL-C, and HOMA-IR were log10-transformed to bring their distributions closer to normal and used in subsequent analyses. A one-way analysis of variance was used to compare baseline characteristics between men and women. We conducted simple linear regression models to examine the association of meal timing (i.e., eating window, caloric midpoint, eating jetlag, time from midsleep point to first food intake, and time from last food intake to midsleep point) with anthropometry (i.e., weight, BMI and waist circumference), body composition (i.e., fat mass percentage, lean mass index, and VAT mass) and cardiometabolic risk factors (i.e., mean blood pressure, triglycerides, total cholesterol, HDL-C, LDL-C, HOMA-IR, and cardiometabolic risk score). We also conducted multiple linear regression models to examine these associations adjusting for potential confounders (i.e., sex, a priori Mediterranean diet pattern, light physical activity, midsleep point or sleep duration, and BMI). Differences between breakfast skippers and consumers in anthropometry, body composition, cardiometabolic risk factors and potential confounders were determined by the Welch's *t* test. Some statistical differences were observed; therefore, additional analyses were performed. Concretely, the simple linear regression models were repeated to examine the association of meal timing with anthropometry, body composition and cardiometabolic risk factors only in breakfast consumers.

The main analyses were corrected for multiple comparison errors (familywise error rate [Hochberg procedure]) [38]. The Statistical Package for the Social Sciences (SPSS) v.25.0 (IBM Corporation, Chicago, IL, USA) was used for all analyses. GraphPad Prism 8 (GraphPad Software, San Diego, CA, USA) was used for plots. Significance was set at *p* < 0.05.

## Results

Table 1 shows the main characteristics of the study participants.

**Table 1:** Descriptive characteristics of participants

	All		Men		Women	
Age (years)	118	22.2 (2.2)	36	22.5 (2.3)	82	22.1 (2.1)
Height (cm)	116	168.0 (8.5)*	34	176.3 (6.4)	82	164.5 (6.7)
Weight (kg)	116	71.2 (16.8)*	34	85.5 (18.5)	82	65.2 (11.7)
*Body composition*						
Body mass index (kg/m^2^)	116	25.1 (4.6)*	34	27.4 (5.4)	82	24.1 (3.9)
Fat mass (kg)	116	25.6 (9.0)	34	27.0 (11.6)	82	25.0 (7.6)
Fat mass (%)	116	36.4 (7.3)*	34	31.1 (7.7)	82	38.6 (5.9)
Lean mass (kg)	116	41.6 (9.8)*	34	53.7 (7.5)	82	36.6 (5.0)
Lean mass index (kg/m^2^)	116	14.6 (2.4)*	34	17.3 (2.2)	82	13.5 (1.4)
Visceral adipose tissue mass (g)	116	346.0 (187.3)*	34	446.7 (188.7)	82	304.3 (171.2)
Waist circumference (cm)	114	80.8 (14.5)*	34	91.4 (16.4)	80	76.3 (10.9)
*Cardiometabolic risk factors*						
Systolic blood pressure (mmHg)	116	116.2 (12.4)*	36	126.0 (12.5)	80	111.8 (9.6)
Diastolic blood pressure (mmHg)	116	70.7 (7.9)*	36	73.0 (10.1)	80	69.7 (6.6)
Mean blood pressure (mmHg)	116	85.9 (8.1)*	36	90.7 (8.8)	80	83.7 (6.7)
Triglycerides (mg/dl)	117	70.0 (52.5, 97.5)	36	74.0 (57.8, 105.8)	81	68.0 (52.0, 89.5)
Total cholesterol (mg/dl)	117	158.0 (143.0, 179.0)	36	153.0 (140.0, 174.8)	81	164.0 (143.0, 183.5)
High-density lipoprotein cholesterol (mg/dl)	117	51.0 (45.5, 58.0)*	36	46.5 (39.3, 51.0)	81	53.0 (47.5, 63.0)
Low-density lipoprotein cholesterol (mg/dl)	117	92.0 (78.5, 109.0)	36	91.0 (81.3, 108.8)	81	92.0 (75.5, 109.0)
Glucose (mg/dl)	117	87.0 (83.0, 92.0)*	36	89.5 (82.3, 97.0)	81	86.0 (83.0, 90.0)
Insulin (μIU/ml)	117	7.2 (5.5, 10.1)	36	7.3 (5.2, 11.4)	81	7.1 (5.5, 9.8)
HOMA-IR	117	1.5 (1.1, 2.2)*	36	1.6 (1.2, 2.5)	81	1.5 (1.1, 2.1)
*Chronobiology*						
Sleep onset (hh:mm)^a^	113	01:13 (01:13)	34	01:15 (01:20)	79	01:12 (01:10)
Sleep offset (hh:mm)^a^	113	08:52 (01:02)	34	09:00 (01:12)	79	08:48 (00:57)
Sleep duration (h)^b^	113	7.85 (1.2)	34	7.98 (1.25)	79	7.8 (1.17))
Midsleep point (hh:mm)^a^	113	05:04 (01:05)	34	05:10 (01:15)	79	05:02 (01:00)
Social jetlag (h)^b^	113	1.42 (1.23)	34	1.40 (1.10)	79	1.42 (1.30)
*Meal timing*						
Eating window (h)^b^	117	12.10 (1.5)	36	12.00 (1.70)	81	12.20 (1.40)
Caloric midpoint (hh:mm)^a^	117	15:54 (01:36)	36	15:48 (01:36)	81	15:54 (01:42)
Eating jetlag (h)^b^	117	1.20 (1.10)	36	1.20 (1.33)	81	1.20 (1.00)
Time from midsleep point to first food intake (h)^b^	113	4.87 (1.40)	34	4.87 (1.72)	79	4.87 (1.23)
Time from last food intake to midsleep point (h)^b^	113	7.05 (1.08)	34	7.22 (1.23)	79	6.97 (1.02)

Data are presented as sample size, mean (standard deviation) when normally distributed, or medians (interquartile range) when not

*HOMA-IR* homeostasis model assessment of insulin resistance

*p ≤ 0.05 for sex comparisons by a one-way analysis of variance (all cardiometabolic risk factors except for blood pressure variables were log10-transformed to bring their distributions closer to normal)

^a^Time shown in local time

^b^Time shown in decimal format

### Meal timing and anthropometry/body composition

The caloric midpoint was negatively associated with fat mass percentage in men (*R*^2^ = 0.089, *β* = − 0.342, *p* = 0.048; Table 2), but became non-significant after adjusting for multiplicity (*p* > 0.05). No further associations were found between meal timing and anthropometry/body composition (all *p* ≥ 0.068; Table 2).

**Table 2:** Association of meal timing with body composition in young adults

Body composition	All				Men				Women			
	*N*	*R*^2^	*β*	*p* value	*N*	*R*^2^	*β*	*p* value	*N*	*R*^2^	*β*	*p* value
*Body mass index (kg/m*^*2*^*)*												
Eating window (h)	116	− 0.004	− 0.070	0.452	34	0.030	− 0.243	0.166	82	− 0.005	0.088	0.433
Caloric midpoint (h)	116	− 0.008	0.029	0.759	34	− 0.009	− 0.146	0.410	82	0.008	0.143	0.201
Eating jetlag (h)	116	− 0.009	− 0.007	0.945	34	− 0.026	− 0.073	0.681	82	− 0.012	0.012	0.917
Time from midsleep point to first food intake (h)	113	− 0.001	0.091	0.339	34	0.030	0.243	0.166	79	− 0.012	− 0.024	0.833
Time from last food intake to midsleep point (h)	113	− 0.008	− 0.032	0.737	34	− 0.031	− 0.010	0.956	79	− 0.001	− 0.110	0.334
*Fat mass (%)*												
Eating window (h)	116	− 0.009	0.003	0.979	34	− 0.015	− 0.125	0.482	82	− 0.012	0.016	0.886
Caloric midpoint (h)	116	− 0.007	− 0.037	0.691	34	0.089	− 0.342	**0.048**	82	− 0.006	0.083	0.460
Eating jetlag (h)	116	− 0.006	− 0.054	0.563	34	− 0.031	− 0.013	0.943	82	− 0.009	− 0.055	0.625
Time from midsleep point to first food intake (h)	113	− 0.006	0.053	0.578	34	− 0.013	0.131	0.459	79	− 0.013	0.005	0.968
Time from last food intake to midsleep point (h)	113	− 0.002	− 0.082	0.390	34	− 0.031	− 0.014	0.938	79	− 0.010	− 0.054	0.634
*Lean mass index (kg/m*^*2*^*)*												
Eating window (h)	116	0.001	− 0.099	0.289	34	0.072	− 0.316	0.068	82	0.004	0.128	0.250
Caloric midpoint (h)	116	− 0.007	0.041	0.659	34	− 0.031	0.022	0.901	82	0.006	0.135	0.228
Eating jetlag (h)	116	− 0.008	0.027	0.776	34	− 0.023	− 0.087	0.625	82	− 0.010	0.052	0.642
Time from midsleep point to first food intake (h)	113	− 0.003	0.074	0.434	34	0.054	0.287	0.100	79	− 0.010	− 0.052	0.651
Time from last food intake to midsleep point (h)	113	− 0.008	0.034	0.724	34	− 0.030	0.029	0.873	79	0.002	− 0.120	0.292
*Visceral adipose tissue mass (g)*												
Eating window (h)	116	0.004	− 0.111	0.236	34	0.037	− 0.257	0.142	82	− 0.012	0.002	0.989
Caloric midpoint (h)	116	− 0.008	0.027	0.771	34	− 0.014	− 0.130	0.464	82	0.000	0.112	0.315
Eating jetlag (h)	116	− 0.006	− 0.049	0.600	34	− 0.030	− 0.034	0.848	82	− 0.005	− 0.087	0.440
Time from midsleep point to first food intake (h)	113	0.002	0.107	0.261	34	0.051	0.282	0.106	79	− 0.013	0.006	0.960
Time from last food intake to midsleep point (h)	113	− 0.009	0.002	0.982	34	− 0.029	− 0.046	0.798	79	− 0.012	− 0.028	0.809
*Waist circumference (cm)*												
Eating window (h)	114	− 0.001	− 0.088	0.352	34	0.049	− 0.279	0.111	80	− 0.001	0.109	0.337
Caloric midpoint (h)	114	− 0.008	− 0.028	0.770	34	0.020	− 0.224	0.203	80	− 0.003	0.101	0.375
Eating jetlag (h)	114	− 0.005	− 0.065	0.491	34	− 0.022	− 0.095	0.594	80	− 0.002	− 0.105	0.352
Time from midsleep point to first food intake (h)	111	− 0.004	0.072	0.450	34	0.007	0.192	0.277	77	− 0.013	− 0.018	0.876
Time from last food intake to midsleep point (h)	111	− 0.009	0.016	0.866	34	− 0.019	0.110	0.536	77	0.008	− 0.147	0.203

Adjusted *R*^2^, *β* standardized regression coefficients and *p* values are obtained from single linear regressions. Neither association remained statistically significant after applying false discovery rate correction (Benjamini–Hochberg)

Bold font is used to indicate statistically significant *p*-values (*p* < 0.05)

### Meal timing and cardiometabolic risk factors

**Fig. 1:**
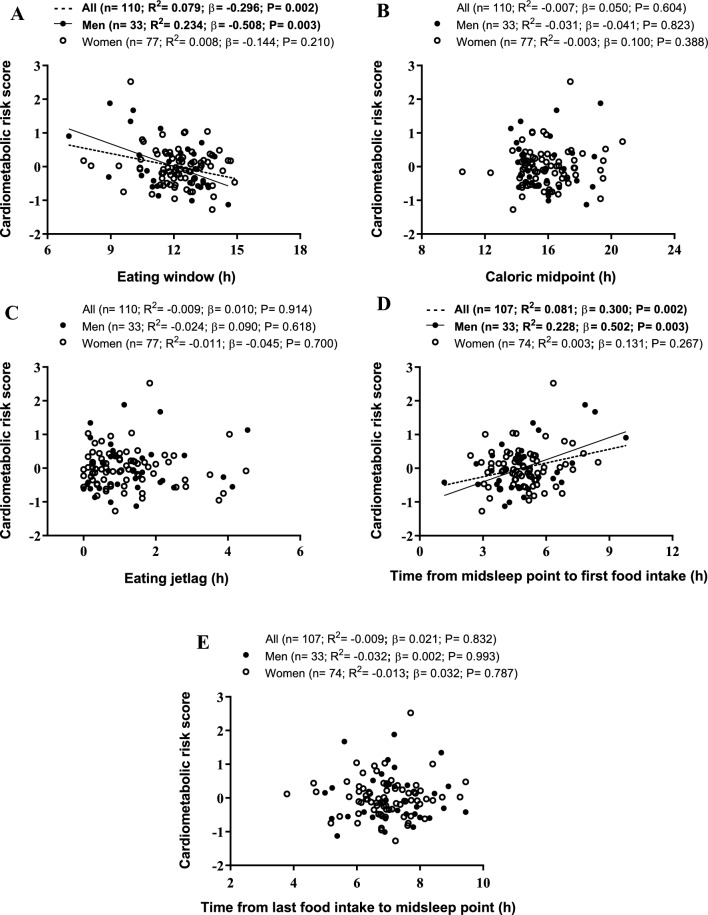
Scatterplots of the associations of meal timing with cardiometabolic risk score (calculated for each sex based on waist circumference, blood pressure, plasma glucose, high-density lipoprotein cholesterol, and triglyceride concentrations, see methods for further details) in young adults. Adjusted *R*^2^, *β* standardized regression coefficients and *p* values are obtained from single linear regressions

The eating window was negatively related to HOMA-IR in all participants and in men (*R*^2^ = 0.100, *β* = − 0.328; *R*^2^ = 0.348, *β* = − 0.605, respectively; all *p* ≤ 0.001; Table 3, Fig. 1 and S2). The eating window was also negatively associated with cardiometabolic risk score in all participants and in men (*R*^2^ = 0.079, *β* = − 0.296; *R*^2^ = 0.234, *β* = − 0.508, respectively; all *p* ≤ 0.003; Table 3, Fig. 1 and S2). A negative association between the eating window and triglycerides was observed in all participants (*R*^2^ = 0.044, *β* = − 0.229, *p* = 0.013; Table 3 and figure S2). The eating window was positively related to HDL-C in all participants and in men (*R*^2^ = 0.048, *β* = 0.238; *R*^2^ = 0.162, *β* = 0.431, respectively; all *p* ≤ 0.010; Table 3 and figure S2). No associations were found between caloric midpoint and eating jetlag with cardiometabolic risk factors (all *p* ≥ 0.150; Table 3). The time from midsleep point to first food intake was positively related to HOMA-IR in all participants and in men (*R*^2^ = 0.078, *β* = 0.294; *R*^2^ = 0.212, *β* = 0.485, respectively; all *p* ≤ 0.003; Table 3, Fig. 1 and S2). The time from midsleep point to first food intake was also positively associated with cardiometabolic risk score in all participants and in men (*R*^2^ = 0.081, *β* = 0.300; *R*^2^ = 0.228, *β* = 0.502, respectively; all *p* ≤ 0.003; Table 3, Fig. 1 and S2). A positive association between the time from midsleep point to first food intake and mean blood pressure was found in all participants (*R*^2^ = 0.030, *β* = 0.197, *p* = 0.037; Table 3 and figure S2). The time from midsleep point to first food intake was negatively associated with HDL-C in all participants and in men (*R*^2^ = 0.045, *β* = − 0.231; *R*^2^ = 0.137, *β* = − 0.403, respectively; all *p* ≤ 0.016; Table 3 and figure S2). A significant positive association between the time from last food intake to midsleep point and triglycerides was found in women (*R*^2^ = 0.087, *β* = 0.314, *p* = 0.005; Table 3). Several variables of meal timing were significantly associated with potential confounders such as a priori Mediterranean diet pattern, light physical activity and midsleep point (all *p* ≤ 0.05; figure S3). Of note is that only the associations of eating window and time from midsleep point to first food intake with HOMA-IR and cardiometabolic risk score—in all participants and men—remained after adjusting for potential confounders and false discovery rate (all *p* ≤ 0.011; Table 2, 3 and S2–S3).

**Table 3:** Association of meal timing with cardiometabolic risk factors in young adults

Cardiometabolic risk factors	All				Men				Women			
	*N*	*R*^2^	*β*	*P* value	*N*	*R*^2^	*β*	*P* value	*N*	*R*^2^	*β*	*P* value
*Mean blood pressure (mmHg)*												
Eating window (h)	116	0.015	− 0.153	0.100	36	0.051	− 0.280	0.099	80	− 0.011	− 0.036	0.749
Caloric midpoint (h)	116	− 0.006	0.054	0.562	36	− 0.007	0.149	0.387	80	− 0.012	0.033	0.773
Eating jetlag (h)	116	− 0.009	− 0.015	0.871	36	− 0.029	− 0.013	0.940	80	− 0.010	− 0.056	0.624
Time from midsleep point to first food intake (h)	112	0.030	0.197	**0.037**	35	0.031	0.245	0.156	77	0.021	0.185	0.107
Time from last food intake to midsleep point (h)	112	− 0.006	− 0.052	0.585	35	− 0.030	0.006	0.973	77	0.017	− 0.174	0.129
*Triglycerides*												
Eating window (h)	117	0.044	− 0.229	**0.013**	36	0.040	− 0.259	0.127	81	0.030	− 0.204	0.068
Caloric midpoint (h)	117	− 0.008	0.032	0.730	36	− 0.005	0.154	0.371	81	− 0.012	− 0.022	0.844
Eating jetlag (h)	117	− 0.005	0.063	0.502	36	0.032	0.245	0.150	81	− 0.010	− 0.051	0.650
Time from midsleep point to first food intake (h)	113	0.005	0.119	0.209	35	0.072	0.315	0.066	78	− 0.012	− 0.026	0.823
Time from last food intake to midsleep point (h)	113	0.015	0.154	0.103	35	− 0.017	− 0.113	0.516	78	0.087	0.314	**0.005**
*Total cholesterol*												
Eating window (h)	117	− 0.006	− 0.052	0.577	36	− 0.012	− 0.130	0.451	81	− 0.012	− 0.017	0.878
Caloric midpoint (h)	117	− 0.009	− 0.013	0.892	36	− 0.020	0.096	0.579	81	− 0.007	− 0.073	0.516
Eating jetlag (h)	117	− 0.001	0.088	0.346	36	− 0.009	0.142	0.409	81	− 0.009	0.058	0.604
Time from midsleep point to first food intake (h)	113	− 0.005	0.063	0.505	35	0.057	0.291	0.090	78	− 0.003	− 0.101	0.380
Time from last food intake to midsleep point (h)	113	− 0.009	0.008	0.936	35	− 0.003	− 0.164	0.347	78	0.007	0.141	0.218
*High-density lipoprotein cholesterol*												
Eating window (h)	117	0.048	0.238	**0.010**	36	0.162	0.431	**0.009**	81	0.008	0.141	0.208
Caloric midpoint (h)	117	− 0.006	0.054	0.560	36	0.013	0.202	0.237	81	− 0.012	− 0.021	0.855
Eating jetlag (h)	117	− 0.009	0.004	0.964	36	0.004	− 0.179	0.296	81	− 0.003	0.099	0.379
Time from midsleep point to first food intake (h)	113	0.045	− 0.231	**0.014**	35	0.137	− 0.403	**0.016**	78	0.021	− 0.184	0.106
Time from last food intake to midsleep point (h)	113	− 0.008	− 0.027	0.774	35	− 0.030	− 0.007	0.966	78	− 0.011	0.041	0.721
*Low-density lipoprotein cholesterol*												
Eating window (h)	117	− 0.001	− 0.088	0.345	36	− 0.001	− 0.167	0.329	81	− 0.011	− 0.039	0.730
Caloric midpoint (h)	117	− 0.008	− 0.020	0.830	36	− 0.029	− 0.008	0.963	81	− 0.012	− 0.027	0.812
Eating jetlag (h)	117	0.000	0.094	0.313	36	− 0.003	0.161	0.349	81	− 0.010	0.053	0.637
Time from midsleep point to first food intake (h)	113	0.011	0.140	0.139	35	0.077	0.323	0.058	78	− 0.013	0.007	0.953
Time from last food intake to midsleep point (h)	113	− 0.007	− 0.041	0.665	35	− 0.008	− 0.146	0.401	78	− 0.012	0.032	0.784
*HOMA-IR*												
Eating window (h)	117	0.100	− 0.328	< 0.001*	36	0.348	− 0.605	< 0.001*	81	− 0.002	− 0.101	0.369
Caloric midpoint (h)	117	− 0.002	0.079	0.399	36	− 0.029	0.015	0.935	81	0.005	0.131	0.245
Eating jetlag (h)	117	− 0.002	0.080	0.393	36	− 0.022	0.085	0.623	81	− 0.007	0.076	0.499
Time from midsleep point to first food intake (h)	113	0.078	0.294	0.002*	35	0.212	0.485	**0.003**	78	0.003	0.126	0.271
Time from last food intake to midsleep point (h)	113	− 0.006	0.053	0.579	35	− 0.017	0.115	0.511	78	− 0.013	− 0.019	0.867
*Cardiometabolic risk score*												
Eating window (h)	110	0.079	− 0.296	0.002*	33	0.234	− 0.508	0.003*	77	0.008	− 0.144	0.210
Caloric midpoint (h)	110	− 0.007	0.050	0.604	33	− 0.031	− 0.041	0.823	77	− 0.003	0.100	0.388
Eating jetlag (h)	110	− 0.009	0.010	0.914	33	− 0.024	0.090	0.618	77	− 0.011	− 0.045	0.700
Time from midsleep point to first food intake (h)	107	0.081	0.300	0.002*	33	0.228	0.502	0.003*	74	0.003	0.131	0.267
Time from last food intake to midsleep point (h)	107	− 0.009	0.021	0.832	33	− 0.032	0.002	0.993	74	− 0.013	0.032	0.787

Adjusted *R*^2^, *β* standardized regression coefficients and *p* values are obtained from single linear regressions. All cardiometabolic risk factors (except for mean blood pressure and cardiometabolic risk score) were log10-transformed to bring their distributions closer to normal. Cardiometabolic risk score was calculated for each sex based on waist circumference, blood pressure, plasma glucose, high-density lipoprotein cholesterol, and triglyceride concentrations (see methods for further details). Symbol * these associations remained statistically significant after applying false discovery rate correction (Benjamini–Hochberg)

Bold font is used to indicate statistically significant *p*-values (*p* < 0.05)

*HOMA-IR* homeostasis model assessment of insulin resistance

### Differences between breakfast skipper and consumer

Significant differences between breakfast skipper and consumer were found for fasting blood glucose, midsleep point, dietary patterns and quality, eating window, caloric midpoint, eating jetlag, and time from midsleep point to first food intake (all *p* ≤ 0.045; table S4). Breakfast consumption influenced the relationship between meal timing and body composition in women observing that the eating window was positively associated with BMI and VAT mass in women breakfast consumers (all *p* ≤ 0.036; figure S4). Breakfast consumption did not modify the relationship shown by meal timing with cardiometabolic risk factors (figure S5).

## Discussion

Our findings suggest that meal timing is not related to anthropometry or body composition parameters in young adults. Similarly, caloric midpoint, eating jetlag and the time from last food intake to midsleep point are not associated with cardiometabolic risk factors. On the other hand, our results show that a longer daily eating window and a shorter time from midsleep point to first food intake (i.e., earlier first food intake in a 24 h cycle) are associated with a healthier cardiometabolic profile (i.e., lower HOMA-IR and cardiometabolic risk score) in young men. These results refute our previous hypothesis, by which a shorter eating window would be associated with a healthier status. However, these findings confirm previous evidence that eating early in alignment with circadian rhythms may play an important role in cardiometabolic health.

The habitual daily eating window in adults in modern societies is ≥ 12 h which is abnormal from an evolutionary perspective [4, 31]. The eating pattern of our hunter-gatherer ancestors was characterized by eating sporadically with inter-meal intervals that depend upon the availability of food sources which included extended fasting period [4]. Moreover, clinical trials have also revealed that reducing the daily eating window results in body weight loss and improvement in cardiometabolic health in adults with overweight or obesity [20–22]. Therefore, we hypothesized that a shorter daily eating window would be associated with better body composition and cardiometabolic health in our cohort of young adults. However, interpreting the cross-sectional relationship between meal timing and health status is a complex task, as it can be influenced by several factors. Indeed, contradictory findings have been reported [9–14, 17, 23, 31, 39, 40]. For instance, we found that the daily eating window is not related to body composition, which could be partly explained by the low variability (standard deviation of 1.5 h) of the daily eating window in our sample. Our results concur with other studies in young adults [12], middle-aged adults [31] and adults with prediabetes [39]. In contrast, others reported that a longer daily eating window is related to a lower BMI [40] and lower fat mass percentage in adults [11]. A confounding factor may be the consumption of breakfast; epidemiological data have constantly shown that breakfast consumption is associated with lower BMI and adiposity [41–43]. Greater energy intake, lower diet quality, lower physical activity levels and misalignment of circadian rhythms are thought to be the physiological mechanisms behind these associations [41–43]. In our cohort of young adults, we observed that adherence to the Mediterranean diet and Mediterranean diet quality was lower in breakfast skippers than in consumers in women. Furthermore, breakfast consumption influences the relationship between meal timing and body composition in women. Specifically, a longer daily eating window was associated with worst body composition (i.e., higher BMI and VAT mass) in women breakfast consumers which is in line with our previous hypothesis and with other study findings in middle-aged women [16] and in older adults [14].

The daily eating window does not take into account the time distribution of food intake, which may be a more important factor than its duration. Indeed, Xiao et al. [14] observed that when a short daily eating window occurs early in the day is associated with a reduced likelihood of being overweight or obese in older adults; while, when a short daily eating window occurs late in the day is related to a higher likelihood of being overweight or obese. For this reason, other meal timing variables have been proposed. One of them is the caloric midpoint, that links meal timing to clock time. A study conducted in Spanish middle-aged adults found that late eaters (i.e., caloric midpoint after 3 pm) had higher BMI, fat mass percentage and waist circumference than early eaters [9], findings that partially concur with those observed in Brazilian young adults [13]. In our study, the caloric midpoint was negatively associated with fat mass percentage in men but became non-significant after adjusting for multiplicity. Another meal-timing variable is eating jetlag, which indicates the variability of the eating midpoint between working and non-working days, being informative about the circadian misalignment. For example, Zerón-Rugerio et al. [17] showed that a greater eating jet lag is related to higher BMI in Spanish and Mexican young adults. Conversely, we observed no association between eating jetlag and body composition in our cohort of Spanish young adults.

Nonetheless, the caloric midpoint and eating jetlag use clock time to illustrate the timing of food intake, which fails to correctly characterize meal timing in relation to the internal circadian time [10, 23]. In this sense, McHill et al. [12] found that a long time from caloric midpoint to melatonin onset is associated with lower BMI and fat mass percentage. However, obtaining measures of dim-light melatonin is not practical in large clinical trials; thus, it has been proposed to measure the timing of food intake relative to the sleep/wake cycle as a proxy for circadian time [10, 23, 24]. Using this methodology, two studies observed that a longer time period from dinner to midsleep point or bedtime is associated with lower adiposity and BMI, respectively [10, 23]. In contrast, we found no relationship between the time from the midsleep point to first food intake or time from last food intake to midsleep point with anthropometry or body composition parameters, which concurs with another study [14]. These discrepancies could be partially explained by the sample size and the assessment of meal and sleep timing.

Regarding cardiometabolic health, we found that a longer daily eating window and a shorter time from midsleep point to first food intake are associated with healthier cardiometabolic profile in men, which agree with previous studies. Concretely, a longer daily eating window has been related to decreased insulin, total cholesterol, LDL-C and increased HDL-C in middle-aged adults [44]. In addition, in concordance with our results, previous studies have found that late eating is related to worst cardiometabolic health [9–16]. Circadian misalignment between the central clock (controlled by external light) and peripheral clocks (regulated by eating, physical activity and sleep among other factors) is one of the mechanisms that may explain these results [6, 7]. In addition, glucose tolerance is higher in the biological morning, which appear to be driven by diurnal variations in β-cell responsiveness, peripheral insulin sensitivity, insulin clearance, and glucose effectiveness [6, 7]. Skeletal muscle fatty acid oxidation and the thermic effect of food are also higher in the biological morning or around noon, which implicates earlier in the daytime is optimal for eating whereas night time is better for fasting and sleep [6, 7]. Our findings could also be partly explained by the consumption of breakfast; epidemiological data have systematically reported that breakfast skipping (i.e., shorter and later daily eating window) is associated with worst cardiometabolic health [41, 42]. As mentioned above, this association could be driven in part by lower diet quality and lower physical activity levels [41–43]. Indeed, in our cohort of young men, we observed that breakfast skippers had a more inflammatory diet and tended to be less physically active. Lastly, others [16] and we did not observe a relationship between meal timing and cardiometabolic risk factors in women, which is intriguing and requires further investigation.

Our findings should be interpreted with caution because the current study suffers from several limitations. Firstly, the cross-sectional design prevents establishing causal relationships. Secondly, we measured the clock timing of food intake via three non-consecutive 24 h recalls. Thirdly, the study population comprised young and healthy adults, limiting the generalizability of the results to older or metabolically compromised individuals. Lastly, the statistical power of the study may have been insufficient to comprehensively investigate potential sex differences in the relationship between meal timing with body composition and cardiometabolic risk factors. Despite these limitations, this study is one of the pioneers in investigating the relationship between meal timing (characterized relative to the sleep/wake cycle) and cardiometabolic risk factors. Further well-designed long-term prospective studies and randomized controlled trials are needed to elucidate the effects of meal timing on body composition and cardiometabolic health.

## Conclusion

Meal timing is not related to either anthropometry or body composition parameters in young adults. Similarly, caloric midpoint, eating jetlag and the time from last food intake to midsleep point are not associated with cardiometabolic risk factors. Nonetheless, a longer daily eating window and a shorter time from midsleep point to first food intake are associated with better cardiometabolic health in men. These results confirm previous evidence that eating early in alignment with circadian rhythms may improve cardiometabolic health. Nutrition strategies aimed to improve cardiometabolic health may contemplate advancing the timing of food intake. Further well-designed studies are needed to confirm these findings and unravel potential mechanisms.

## Supplementary Information

Below is the link to the electronic supplementary material.

Supplementary file1 (PDF 891 KB)

## Data Availability

The deidentified participant data that support the findings of this study are available from the corresponding author upon reasonable request.
