# Correction to: Association of meal timing with body composition and cardiometabolic risk factors in young adults

**DOI:** 10.1007/s00394-023-03175-z

**Published:** 2023-06-01

**Authors:** Manuel Dote-Montero, Francisco M. Acosta, Guillermo Sanchez-Delgado, Elisa Merchan-Ramirez, Francisco J. Amaro-Gahete, Idoia Labayen, Jonatan R. Ruiz

**Affiliations:** 1 Department of Physical Education and Sports, Faculty of Sports Science, Sport and Health University Research Institute (iMUDS), University of Granada, Carretera de Alfacar s/n, 18071 Granada, Spain; 2 Turku PET Centre, University of Turku, Turku, Finland; 3 Turku PET Centre, Turku University Hospital, Turku, Finland; 4 InFLAMES Research Flagship Center, University of Turku, Turku, Finland; 5 Division of Endocrinology, Department of Medicine, Centre de Recherche du Centre Hospitalier Universitaire de Sherbrooke, Université de Sherbrooke, Sherbrooke, QC Canada; 6 CIBER de Fisiopatología de la Obesidad y Nutrición (CIBEROBN), Instituto de Salud Carlos III, Granada, Spain; 7 Institute for Sustainability and Food Chain Innovation (ISFOOD), University of Navarra, Pamplona, Spain; 8 Navarra Institute for Health Research, IdiSNA, Pamplona, Spain; 9 Department of Health Sciences, Public University of Navarra, Campus de Arrosadia, Pamplona, Spain; 10 Instituto de Investigación Biosanitaria, Ibs.Granada, Granada, Spain

**Correction to:** **European Journal of Nutrition** 10.1007/s00394-023-03141-9

The original version of this article unfortunately contained a mistake. The x-axis visualization is somewhat blurred or diffused in Fig. 1.

The corrected Fig. 1 is given in the next page.

**Fig. 1:**
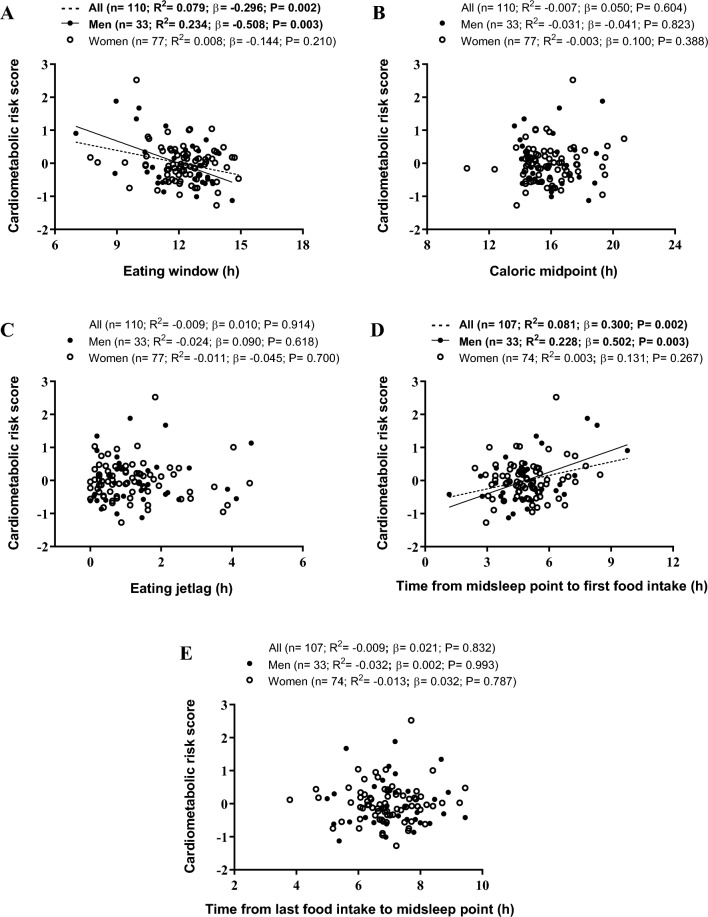
Scatterplots of the associations of meal timing with cardiometabolic risk score (calculated for each sex based on waist circumference, blood pressure, plasma glucose, high-density lipoprotein cholesterol, and triglyceride concentrations, see methods for further details) in young adults. Adjusted *R*^2^, *β* standardized regression coefficients and *p* values are obtained from single linear regressions

The original article has been corrected.

