# Supplementary material for: Association of meal timing with body composition and cardiometabolic risk factors in young adults
Source: Eur J Nutr. 2023 Apr 26;62(5):2303–15. doi: 10.1007/s00394-023-03141-9 (PMC10349779; doi:10.1007/s00394-023-03141-9)
Supplement: Supplementary file 1 — Supplementary file1 (PDF 891 KB) [file 394_2023_3141_MOESM1_ESM.pdf]

Association of meal timing with body composition and cardiometabolic risk factors in young adults

European Journal of Nutrition

Authors

Manuel Dote-Montero[a], Francisco M. Acosta[a,b,c,d], Guillermo Sanchez-Delgado[a,e], Elisa Merchan-Ramirez[a], Francisco J. Amaro-Gahete[a,f], Idoia Labayen[g,h,i] and Jonatan R. Ruiz[a,j]

Affiliations

[a]PROmoting FITness and Health through Physical Activity Research Group, Sport and Health University Research Institute, Department of Physical Education and Sports, Faculty of Sport Sciences, University of Granada, Granada, Spain.

[b]Turku PET Centre, University of Turku, Turku, Finland.

[c]Turku PET Centre, Turku University Hospital, Turku, Finland.

[d]InFLAMES Research Flagship Center, University of Turku, Finland.

[e]Department of Medicine, Division of Endocrinology, Centre de Recherche du Centre Hospitalier Universitaire de Sherbrooke, Université de Sherbrooke, Sherbrooke, Quebec, Canada.

[f]EFFECTS-262 Research Group, Department of Physiology, Faculty of Medicine, University of Granada, Granada, Spain.

[g]Institute for Sustainability & Food Chain Innovation (ISFOOD), University of Navarra, Pamplona, Spain.

[h]Navarra Institute for Health Research, IdiSNA, Pamplona, Spain.

[i]Department of Health Sciences, Public University of Navarra, Campus de Arrosadia, Pamplona, Spain.

[j]Instituto de Investigación Biosanitaria, ibs.Granada, Granada, Spain.

Keywords: Chrononutrition; Circadian rhythms; Timing of food intake; Intermittent fasting; Fat mass; Insulin resistence.

Running title: meal timing, body composition and metabolic health

Corresponding authors

Manuel Dote-Montero. Department of Physical and Sports Education, Faculty of Sports Science, University of Granada, Carretera de Alfacar s/n, 18071, Granada, Spain. Email: manueldote@ugr.es

Jonatan Ruiz Ruiz, Department of Physical and Sports Education, Faculty of Sports Science, University of Granada, Carretera de Alfacar s/n, 18071 Granada, Spain. E-mail: ruizj@ugr.es

Table S1. Reporting Table for STROBE-nut: An extension of the STROBE statement for nutritional epidemiology.

Item	Item nr	STROBE recommendations	Extension for Nutritional Epidemiology studies (STROBE-nut)	Reported on page #
**Title and abstract**	1	(a) Indicate the study's design with a commonly used term in the title or the abstract.<br>(b) Provide in the abstract an informative and balanced summary of what was done and what was found.	**nut-1** State the dietary/nutritional assessment method(s) used in the title, abstract, or keywords.	2
**Introduction**				
**Background rationale**	2	Explain the scientific background and rationale for the investigation being reported.		3-4
**Objectives**	3	State specific objectives, including any pre-specified hypotheses.		4
**Methods**				
**Study design**	4	Present key elements of study design early in the paper.		5
**Settings**	5	Describe the setting, locations, and relevant dates, including periods of recruitment, exposure, follow-up, and data collection.	**nut-5** Describe any characteristics of the study settings that might affect the dietary intake or nutritional status of the participants, if applicable.	5
**Participants**	6	a) Cohort study—Give the eligibility criteria, and the sources and methods of selection of participants. Describe methods of follow-up.<br>Case-control study—Give the eligibility criteria, and the sources and methods of case ascertainment and control selection. Give the rationale for the choice of cases and controls.<br>Cross-sectional study—Give the eligibility criteria, and the sources and methods of selection of participants.<br>(b) Cohort study—For matched studies, give matching criteria and number of exposed and unexposed.<br>Case-control study—For matched studies, give matching criteria and the number of controls per case.	**nut-6** Report particular dietary, physiological or nutritional characteristics that were considered when selecting the target population.	5
**Variables**	7	Clearly define all outcomes, exposures, predictors, potential confounders, and effect modifiers. Give diagnostic criteria, if applicable.	**nut-7.1** Clearly define foods, food groups, nutrients, or other food components.<br>**nut-7.2** When using dietary patterns or indices, describe the methods to obtain them and their nutritional properties.	5-8
**Data sources - measurements**	8	For each variable of interest, give sources of data and details of methods of assessment (measurement). Describe comparability of assessment methods if there is more than one group.	**nut-8.1** Describe the dietary assessment method(s), e.g., portion size estimation, number of days and items recorded, how it was developed and administered, and how quality was assured. Report if and how supplement intake was assessed.<br>**nut-8.2** Describe and justify food composition data used. Explain the procedure to match food composition with consumption data. Describe the use of conversion factors, if applicable.<br>**nut-8.3** Describe the nutrient requirements, recommendations, or dietary guidelines and the evaluation approach used to compare intake with the dietary reference values, if applicable.<br>**nut-8.4** When using nutritional biomarkers, additionally use the STROBE Extension for Molecular Epidemiology (STROBE-ME). Report the type of biomarkers used and their usefulness as dietary exposure markers.<br>**nut-8.5** Describe the assessment of nondietary data (e.g., nutritional status and influencing factors) and timing of the assessment of these variables in relation to dietary assessment.<br>**nut-8.6** Report on the validity of the dietary or nutritional assessment methods and any internal or external validation used in the study, if applicable.	5-8
**Bias**	9	Describe any efforts to address potential sources of bias.	**nut-9** Report how bias in dietary or nutritional assessment was addressed, e.g., misreporting, changes in habits as a result of being measured, or data imputation from other sources	-
**Study Size**	10	Explain how the study size was arrived at.		5
**Quantitative variables**	11	Explain how quantitative variables were handled in the analyses. If applicable, describe which groupings were chosen and why.	**nut-11** Explain categorization of dietary/nutritional data (e.g., use of N-tiles and handling of nonconsumers) and the choice of reference category, if applicable.	5-8
**Statistical Methods**	12	(a) Describe all statistical methods, including those used to control for confounding<br>(b) Describe any methods used to examine subgroups and interactions.<br>(c) Explain how missing data were addressed.	**nut-12.1** Describe any statistical method used to combine dietary or nutritional data, if applicable.<br>**nut-12.2** Describe and justify the method for energy adjustments, intake modeling, and use of weighting factors, if applicable.	8-9

		(d) Cohort study—If applicable, explain how loss to follow-up was addressed.<br>Case-control study—If applicable, explain how matching of cases and controls was addressed.<br>Cross-sectional study—If applicable, describe analytical methods taking account of sampling strategy.<br>(e) Describe any sensitivity analyses.	**nut-12.3** Report any adjustments for measurement error, i.e,. from a validity or calibration study.	
**Results**				
**Participants**	13	(a) Report the numbers of individuals at each stage of the study—e.g., numbers potentially eligible, examined for eligibility, confirmed eligible, included in the study, completing follow-up, and analyzed.<br>(b) Give reasons for non-participation at each stage.<br>(c) Consider use of a flow diagram.	**nut-13** Report the number of individuals excluded based on missing, incomplete or implausible dietary/nutritional data.	10
**Descriptive data**	14	(a) Give characteristics of study participants (e.g., demographic, clinical, social) and information on exposures and potential confounders<br>(b) Indicate the number of participants with missing data for each variable of interest<br>(c) Cohort study—Summarize follow-up time (e.g., average and total amount)	**nut-14** Give the distribution of participant characteristics across the exposure variables if applicable. Specify if food consumption of total population or consumers only were used to obtain results.	10
**Outcome data**	15	Cohort study—Report numbers of outcome events or summary measures over time.<br>Case-control study—Report numbers in each exposure category, or summary measures of exposure.<br>Cross-sectional study—Report numbers of outcome events or summary measures.		10-11
**Main results**	16	(a) Give unadjusted estimates and, if applicable, confounder-adjusted estimates and their precision (e.g., 95% confidence interval).<br>Make clear which confounders were adjusted for and why they were included.<br>(b) Report category boundaries when continuous variables were categorized.<br>(c) If relevant, consider translating estimates of relative risk into absolute risk for a meaningful time period.	**nut-16** Specify if nutrient intakes are reported with or without inclusion of dietary supplement intake, if applicable.	10-11
**Other analyses**	17	Report other analyses done—e.g., analyses of subgroups and interactions and sensitivity analyses.	**nut-17** Report any sensitivity analysis (e.g., exclusion of misreporters or outliers) and data imputation, if applicable.	11
**Discussion**				
**Key results**	18	Summarize key results with reference to study objectives.		
**Limitation**	19	Discuss limitations of the study, taking into account sources of potential bias or imprecision. Discuss both direction and magnitude of any potential bias.	**nut-19** Describe the main limitations of the data sources and assessment methods used and implications for the interpretation of the findings.	15
**Interpretation**	20	Give a cautious overall interpretation of results considering objectives, limitations, multiplicity of analyses, results from similar studies, and other relevant evidence.	**nut-20** Report the nutritional relevance of the findings, given the complexity of diet or nutrition as an exposure.	15
**Generalizability**	21	Discuss the generalizability (external validity) of the study results.		15
**Other information**				
**Funding**	22	Give the source of funding and the role of the funders for the present study and, if applicable, for the original study on which the present article is based.		1
Ethics			**nut-22.1** Describe the procedure for consent and study approval from ethics committee(s).	5
Supplementary material			**nut-22.2** Provide data collection tools and data as online material or explain how they can be accessed.	5-11

Table S2. Association of meal timing with body composition after adjusting for potential confounders in young adults.

Body composition	P-value														
	All (N=116)					Men (N=34)					Women (N=82)				
	M0	M1	M2	M3	M4	M0	M1	M2	M3	M4	M0	M1	M2	M3	M4
Body mass index (Kg/m^2)															
Eating window (h)	0.452	0.617	0.574	0.516	0.493	0.166	-	0.121	0.116	0.105	0.433	-	0.410	0.293	0.280
Caloric midpoint (h)	0.759	0.662	0.643	0.972	0.811	0.410	-	0.369	0.373	0.412	0.201	-	0.205	0.594	0.353
Eating jetlag (h)	0.945	0.815	0.848	0.780	0.839	0.681	-	0.783	0.787	0.723	0.917	-	0.933	0.855	0.984
Time from midsleep point to first food intake (h)[a]	0.339	0.313	0.277	0.351	0.185	0.166	-	0.111	0.117	**0.015**	0.833	-	0.825	0.531	0.388
Time from last food intake to midsleep point (h)[a]	0.737	0.474	0.482	0.689	0.464	0.956	-	0.925	0.929	0.418	0.334	-	0.334	0.412	0.477
Fat mass (%)															
Eating window (h)	0.979	0.677	0.663	0.665	0.719	0.482	-	0.373	0.493	0.424	0.886	-	0.809	0.766	0.550
Caloric midpoint (h)	0.691	0.535	0.539	0.527	0.816	**0.048**	-	**0.037**	**0.047**	0.063	0.460	-	0.505	0.652	0.333
Eating jetlag (h)	0.563	0.682	0.686	0.688	0.837	0.943	-	0.918	0.949	0.922	0.625	-	0.572	0.520	0.695
Time from midsleep point to first food intake (h)[a]	0.578	0.528	0.512	0.519	0.502	0.459	-	0.332	0.317	0.127	0.968	-	0.972	0.866	0.513
Time from last food intake to midsleep point (h)[a]	0.390	0.691	0.694	0.704	0.702	0.938	-	0.899	0.626	0.302	0.634	-	0.612	0.658	0.965
Lean mass index (Kg/m^2)															
Eating window (h)	0.289	0.514	0.462	0.380	0.330	0.068	-	0.058	**0.029**	**0.032**	0.250	-	0.268	0.160	0.234
Caloric midpoint (h)	0.659	0.334	0.316	0.759	0.713	0.901	-	0.920	0.997	0.979	0.228	-	0.205	0.684	0.570
Eating jetlag (h)	0.776	0.949	0.996	0.897	0.856	0.625	-	0.653	0.681	0.704	0.642	-	0.608	0.820	0.816
Time from midsleep point to first food intake (h)[a]	0.434	0.261	0.222	0.307	0.098	0.100	-	0.082	0.088	**0.009**	0.651	-	0.695	0.387	0.419
Time from last food intake to midsleep point (h)[a]	0.724	0.562	0.572	0.892	0.510	0.873	-	0.884	0.617	0.878	0.292	-	0.306	0.384	0.292
Visceral adipose tissue mass (g)															
Eating window (h)	0.236	0.342	0.312	0.272	0.285	0.142	-	0.077	0.095	0.077	0.989	-	0.926	0.784	0.685
Caloric midpoint (h)	0.771	0.669	0.653	0.997	0.743	0.464	-	0.384	0.418	0.481	0.315	-	0.343	0.821	0.516
Eating jetlag (h)	0.600	0.467	0.485	0.439	0.539	0.848	-	0.959	0.975	0.920	0.440	-	0.402	0.261	0.369
Time from midsleep point to first food intake (h)[a]	0.261	0.233	0.207	0.260	0.178	0.106	-	**0.046**	**0.047**	**0.008**	0.960	-	0.997	0.704	0.548
Time from last food intake to midsleep point (h)[a]	0.982	0.721	0.728	0.951	0.855	0.798	-	0.744	0.602	0.296	0.809	-	0.791	0.923	0.945
Waist circumference (cm)[b]															
Eating window (h)	0.352	0.541	0.525	0.472	0.463	0.111	-	0.077	0.078	0.087	0.337	-	0.293	0.209	0.283
Caloric midpoint (h)	0.770	0.842	0.847	0.570	0.738	0.203	-	0.179	0.187	0.178	0.375	-	0.407	0.808	0.443
Eating jetlag (h)	0.491	0.292	0.292	0.287	0.328	0.594	-	0.687	0.689	0.722	0.352	-	0.317	0.243	0.337
Time from midsleep point to first food intake (h)[a]	0.450	0.393	0.373	0.421	0.188	0.277	-	0.200	0.206	**0.048**	0.876	-	0.838	0.595	0.607
Time from last food intake to midsleep point (h)[a]	0.866	0.722	0.727	0.895	0.564	0.536	-	0.565	0.500	0.989	0.203	-	0.196	0.335	0.175

P values are obtained for Model 0 (single linear regression), then the analyses were adjusted for: sex (only in all, Model 1); sex and *a priori* Mediterranean diet pattern (MeD-P) (Model 2); sex, *a priori* Mediterranean diet pattern and light physical activity (min/day)[c] (Model 3); sex, *a priori* Mediterranean diet pattern, light physical activity (min/day) and midsleep point (h)[d] (Model 4). Sex was included only when men and women were analysed together (i.e., all). In *time from midsleep point to first food intake* and *time from last food intake to midsleep point* model 4 included sleep duration (h) instead of midsleep point. Neither association remained statistically significant after applying false discovery rate correction (Benjamini-Hochberg). Some specific outcomes had missing data for all and women: [a,d]3 missing participants, [b]2 missing participants, and [c]1 missing participant. Abbreviation: M, Model.

Table S3. Association of meal timing with cardiometabolic risk factors after adjusting for potential confounders in young adults.

Cardiometabolic risk factors	P-value																	
	All (N=117)						Men (N=36)						Women (N=81)					
	M0	M1	M2	M3	M4	M5	M0	M1	M2	M3	M4	M5	M0	M1	M2	M3	M4	M5
Mean blood pressure (mmHg)[a]																		
Eating window (h)	0.100	0.140	0.185	0.156	0.160	0.232	0.099	-	0.099	0.118	0.158	0.472	0.749	-	0.902	0.990	0.770	0.554
Caloric midpoint (h)	0.562	0.441	0.489	0.705	0.817	0.925	0.387	-	0.385	0.386	0.421	0.200	0.773	-	0.919	0.533	0.513	0.328
Eating jetlag (h)	0.871	0.684	0.577	0.542	0.330	0.339	0.940	-	0.934	0.890	0.715	0.847	0.624	-	0.503	0.340	0.263	0.235
Time from midsleep point to first food intake (h)[b]	**0.037**	**0.026**	**0.037**	**0.050**	**0.011**	**0.033**	0.156	-	0.166	0.176	0.059	0.333	0.107	-	0.141	0.260	0.147	0.078
Time from last food intake to midsleep point (h)[b]	0.585	0.281	0.260	0.376	0.200	0.289	0.973	-	0.974	0.989	0.696	0.905	0.129	-	0.111	0.183	0.107	0.133
Triglycerides																		
Eating window (h)	**0.013**	**0.015**	**0.014**	**0.014**	**0.032**	**0.044**	0.127	-	0.105	0.166	0.150	0.374	0.068	-	0.074	0.069	0.220	0.149
Caloric midpoint (h)	0.730	0.704	0.705	0.586	0.620	0.636	0.371	-	0.409	0.362	0.314	0.163	0.844	-	0.804	0.810	0.700	0.594
Eating jetlag (h)	0.502	0.508	0.506	0.471	0.450	0.405	0.150	-	0.120	0.140	0.222	0.146	0.650	-	0.617	0.651	0.599	0.611
Time from midsleep point to first food intake (h)[b]	0.209	0.207	0.210	0.178	**0.042**	0.078	0.066	-	**0.047**	**0.044**	**0.012**	0.072	0.823	-	0.777	0.819	0.846	0.723
Time from last food intake to midsleep point (h)[b]	0.103	0.126	0.129	0.155	0.315	0.230	0.516	-	0.506	0.299	0.141	0.221	**0.005**	-	**0.006**	**0.006**	**0.012**	**0.009**
Total cholesterol																		
Eating window (h)	0.577	0.519	0.540	0.564	0.410	0.457	0.451	-	0.423	0.440	0.234	0.544	0.878	-	0.946	0.890	0.951	0.874
Caloric midpoint (h)	0.892	0.848	0.834	0.973	0.769	0.784	0.579	-	0.606	0.601	0.388	0.210	0.516	-	0.470	0.635	0.671	0.724
Eating jetlag (h)	0.346	0.337	0.351	0.337	0.199	0.186	0.409	-	0.377	0.389	0.343	0.239	0.604	-	0.645	0.577	0.469	0.477
Time from midsleep point to first food intake (h)[b]	0.505	0.509	0.210	0.469	0.288	0.356	0.090	-	0.062	0.063	**0.039**	0.210	0.380	-	0.346	0.410	0.510	0.455
Time from last food intake to midsleep point (h)[b]	0.936	0.800	0.129	0.901	0.898	0.952	0.347	-	0.337	0.252	0.184	0.288	0.218	-	0.234	0.260	0.322	0.347
High-density lipoprotein cholesterol																		
Eating window (h)	**0.010**	**0.011**	**0.012**	**0.012**	**0.007***	**0.009**	**0.009**	-	**0.012**	**0.034**	**0.015**	0.050	0.208	-	0.208	0.217	0.214	0.051
Caloric midpoint (h)	0.560	0.665	0.646	0.713	0.989	0.956	0.237	-	0.272	0.321	0.419	0.580	0.855	-	0.865	0.923	0.763	0.942
Eating jetlag (h)	0.964	0.918	0.875	0.917	0.889	0.971	0.296	-	0.349	0.412	0.669	0.543	0.379	-	0.371	0.345	0.401	0.384
Time from midsleep point to first food intake (h)[b]	**0.014**	**0.006***	**0.006***	**0.005***	**0.008***	**0.020**	**0.016**	-	**0.023**	**0.013**	**0.010**	0.066	0.106	-	0.106	0.120	0.179	**0.046**
Time from last food intake to midsleep point (h)[b]	0.774	0.796	0.783	0.722	0.996	0.764	0.966	-	0.931	0.523	0.538	0.761	0.721	-	0.719	0.757	0.978	0.802
Low-density lipoprotein cholesterol																		
Eating window (h)	0.345	0.344	0.384	0.398	0.212	0.262	0.329	-	0.328	0.377	0.363	0.412	0.730	-	0.814	0.778	0.835	0.787
Caloric midpoint (h)	0.830	0.828	0.799	0.910	0.639	0.663	0.963	-	0.963	0.989	0.697	0.452	0.812	-	0.742	0.845	0.896	0.932
Eating jetlag (h)	0.313	0.315	0.347	0.332	0.173	0.142	0.349	-	0.345	0.367	0.331	0.213	0.637	-	0.693	0.646	0.450	0.448
Time from midsleep point to first food intake (h)[b]	0.139	0.141	0.151	0.132	0.102	0.164	0.058	-	**0.046**	**0.045**	**0.038**	0.235	0.953	-	0.990	0.957	0.932	0.889
Time from last food intake to midsleep point (h)[b]	0.665	0.696	0.686	0.609	0.581	0.667	0.401	-	0.399	0.257	0.248	0.385	0.784	-	0.822	0.844	0.854	0.829
HOMA-IR																		
Eating window (h)	**<0.001***	**<0.001***	**<0.001***	**<0.001***	**0.001***	**0.001***	**<0.001***	-	**<0.001***	**<0.001***	**<0.001***	**0.002**	0.369	-	0.459	0.541	0.659	0.367
Caloric midpoint (h)	0.399	0.372	0.392	0.431	0.553	0.539	0.932	-	**0.042**	0.845	0.915	0.411	0.245	-	0.299	0.737	0.674	0.896
Eating jetlag (h)	0.393	0.398	0.434	0.429	0.520	0.418	0.623	-	0.579	0.670	0.873	0.685	0.499	-	0.573	0.719	0.668	0.615
Time from midsleep point to first food intake (h)[b]	**0.002***	**0.001***	**0.002***	**0.002***	**<0.001***	**0.001***	**0.003**	-	**0.002***	**0.001***	**<0.001***	**0.004**	0.271	-	0.320	0.481	0.340	0.160
Time from last food intake to midsleep point (h)[b]	0.579	0.679	0.699	0.644	0.804	0.493	0.511	-	0.520	0.928	0.871	0.618	0.867	-	0.806	0.930	0.787	0.946
Cardiometabolic risk score[c]																		
Eating window (h)	**0.002***	**0.002***	**0.002***	**0.002***	**0.002***	**<0.001***	**0.003***	-	**0.002***	**0.003***	**0.003***	**0.011**	0.210	-	0.233	0.257	0.347	**0.041**
Caloric midpoint (h)	0.604	0.604	0.619	0.776	0.639	0.654	0.823	-	0.815	0.885	0.963	0.521	0.388	-	0.421	0.852	0.682	0.931
Eating jetlag (h)	0.914	0.926	0.962	0.938	0.889	0.843	0.618	-	0.604	0.687	0.741	0.473	0.700	-	0.655	0.574	0.667	0.531
Time from midsleep point to first food intake (h)[b]	**0.002***	**0.002***	**0.002***	**0.002***	**<0.001***	**<0.001***	**0.003***	-	**0.002***	**0.002***	**<0.001***	**0.006**	0.267	-	0.285	0.464	0.223	**0.018**
Time from last food intake to midsleep point (h)[b]	0.832	0.841	0.857	0.749	0.987	0.638	0.993	-	0.992	0.815	0.589	0.965	0.787	-	0.819	0.584	0.843	0.622

P values are obtained for Model 0 (single linear regression), then analyses were adjusted for: sex (only in all, Model 1); sex and *a priori* Mediterranean diet pattern (MeD-P) (Model 2); sex, *a priori* Mediterranean diet pattern and light physical activity (min/day)[d] (Model 3); sex, *a priori* Mediterranean diet pattern, light physical activity (min/day) and midsleep point (h)[e] (Model 4); sex, *a priori* Mediterranean diet pattern, light physical activity (min/day), midsleep point (h) and body mass index (kg/m^2)[f] (Model 5). Sex was included only when men and women were analysed together (i.e., all). In *time from midsleep point to breakfast* and *time from dinner to midsleep point* model 4 and 5 included sleep duration (h) instead of midsleep point. All cardiometabolic risk factors (except for mean blood pressure and cardiometabolic risk score) were log10-transformed to bring their distributions closer to normal. Cardiometabolic risk score was calculated for each sex based on waist circumference, blood pressure, plasma glucose, high-density lipoprotein cholesterol, and triglyceride concentrations (see methods for further details). Symbol * these associations remained statistically significant after applying false discovery rate correction (Benjamini-Hochberg). Some specific outcomes had missing data for all: [a,d,f]1 missing participant, [b,e]4 missing participants, and [c]7 missing participants; for men: [c]3 missing participants and [e,f]1 missing participant; and for women: [a,d]1 missing participant, [c]4 missing participants and [b,e]3 missing participants. Abbreviations: HOMA-IR, homeostasis model assessment of insulin resistance; M, Model.

Table S4. Differences between breakfast skipper and consumer in body composition, cardiometabolic risk factors, physical activity, sleep patterns, dietary patterns, and energy and macronutrients intake.

	All		Men		Women	
	Skip. (N)= 29; Cons. (N)= 88		Skip. (N)= 10; Cons. (N)= 26		Skip. (N)= 19; Cons. (N)= 62	
	MD (95% CI)	P-value	MD (95% CI)	P-value	MD (95% CI)	P-value
Body mass index (Kg/m^2)[a]	0.7 (-1.7, 3.1)	0.551	1.3 (-4.3, 6.9)	0.619	0.3 (-2.1, 2.7)	0.816
Fat mass (kg)[a]	1.3 (-3.1, 5.7)	0.564	2.4 (-8.2, 13)	0.630	0.6 (-4.1, 5.4)	0.78
Fat mass (%)[a]	-0.3 (-3.6, 3.0)	0.856	0.8 (-5.0, 6.7)	0.768	-0.4 (-4.1, 3.3)	0.810
Lean mass index (Kg m^2)[a]	0.4 (-0.8, 1.6)	0.697	0.6 (-1.7, 3.0)	0.569	0.1 (-0.6, 0.9)	0.897
Visceral adipose tissue mass (g)[a]	23.9 (-73.8, 121.6)	0.494	41.5 (-155.1, 238.2)	0.650	9.0 (-102.0, 120.1)	0.868
Waist circumference (cm)[b]	1.4 (-6.2, 9.1)	0.702	3.7 (-12.8, 20.2)	0.634	-0.6 (-7.6, 6.5)	0.872
Systolic blood pressure (mmHg)[c]	0.9 (-4.6, 6.5)	0.734	0.3 (-10.5, 11.2)	0.948	-0.2 (-4.9, 4.5)	0.926
Diastolic blood pressure (mmHg)[c]	2.6 (-0.9, 6.2)	0.141	4.2 (-3.9, 12.3)	0.284	1.5 (-2.1, 5.2)	0.387
Mean blood pressure (mmHg)[c]	2.1 (-1.7, 5.9)	0.275	2.9 (-5.1, 10.9)	0.445	1.0 (-2.6, 4.5)	0.587
Triglycerides (mg/dl)	19.7 (-12.8, 52.2)	0.227	42.6 (-25.6, 110.8)	0.196	7.5 (-30.4, 45.4)	0.684
Total cholesterol (mg/dl)	7.1 (-9, 23.1)	0.378	24.3 (-8.8, 57.5)	0.136	-0.9 (-19.9, 18.0)	0.920
High-density lipoprotein cholesterol (mg/dl)	-0.5 (-5.7, 4.7)	0.848	-2.9 (-9.2, 3.4)	0.343	1.5 (-4.7, 7.6)	0.629
Low-density lipoprotein cholesterol (mg/dl)	6.5 (-6.3, 19.4)	0.311	20.3 (-3.2, 43.7)	0.085	-0.3 (-16.2, 15.6)	0.968
Glucose (mg/dl)	5.2 (1.9, 8.4)	**0.003**	9.1 (2.2, 16.0)	**0.014**	3.0 (-0.1, 6.0)	0.06
Insulin (µIU/ml)	3.5 (-0.3, 7.3)	0.068	8.3 (-2.2, 18.7)	0.108	1.0 (-1.4, 3.3)	**0.045**
HOMA-IR	1.0 (0.0, 2.0)	0.057	2.4 (-0.6, 5.3)	0.100	0.3 (-0.3, 0.9)	0.324
Cardiometabolic risk score[d]	0.2 (-0.1, 0.6)	0.233	0.5 (-0.2, 1.3)	0.150	0.1 (-0.4, 0.5)	0.792
Physical activity (ENMO/day)[e]	0.1 (-1.8, 2.0)	0.924	-2.7 (-5.5, 0.2)	0.066	1.5 (-0.8, 3.9)	0.196
Sedentary time (min/day)[e]	-7.3 (-33.1, 18.5)	0.571	3 (-35.5, 41.5)	0.875	-13.8 (-48.4, 20.7)	0.419
Cardiorespiratory fitness (ml/kg/min)	1.1 (-3.1, 5.2)	0.604	-0.6 (-9.1, 8.0)	0.894	1.5 (-3.2, 6.3)	0.517
Sleep duration (h)[f]	0.0 (-0.5, 0.5)	0.866	0.3 (-0.6, 1.2)	0.524	-0.2 (-0.8, 0.4)	0.427
Midsleep point (h)[f]	0.4 (-0.1, 1.0)	0.102	0 (-1.1, 1.1)	0.998	0.6 (0.0, 1.3)	**0.045**
Social jet lag (h)[f]	-0.3 (-0.8, 0.2)	0.246	-0.5 (-1.3, 0.2)	0.141	-0.1 (-0.8, 0.5)	0.655
MeD-P	-2.4 (-4.3, -0.5)	**0.013**	-1.5 (-4.8, 1.7)	0.336	-2.7 (-5.1, -0.3)	**0.028**
MeD-S[g]	-0.6 (-1.3, 0.0)	**0.050**	-0.7 (-2.3, 0.9)	0.374	-0.6 (-1.2, 0.0)	0.068
MeD-DQI[h]	1.3 (0.4, 2.3)	**0.007**	1.2 (-0.7, 3)	0.208	1.3 (0.2, 2.4)	**0.024**
DASH[i]	-1.4 (-3.3, 0.4)	0.129	-1.4 (-5.4, 2.5)	0.452	-1.4 (-3.2, 0.4)	0.132
DQI	0.6 (-0.4, 1.6)	0.215	0.5 (-1.3, 2.3)	0.553	0.6 (-0.6, 1.8)	0.330
DII	0.5 (-0.1, 1.1)	0.087	1.2 (0.3, 2.2)	**0.015**	0.2 (-0.6, 1.0)	0.581
Energy intake (kcal/day)	-24.5 (-230.2, 181.2)	0.812	-210.2 (-588.0, 167.6)	0.262	38.6 (-209.6, 286.7)	0.752
Energy density (kcal/g)	0.1 (-0.1, 0.2)	0.542	0.0 (-0.3, 0.3)	0.797	0.1 (-0.1, 0.3)	0.383
Carbohydrates (% energy)	-1.4 (-4.1, 1.2)	0.292	-0.2 (-5.0, 4.6)	0.925	-1.8 (-5.0, 1.5)	0.283
Protein (% energy)	-0.2 (-1.4, 1.0)	0.718	-1.3 (-3.6, 1.0)	0.244	0.2 (-1.2, 1.6)	0.784
Fat (% energy)	2.1 (-0.5, 4.7)	0.104	2.8 (-2.4, 8.1)	0.273	1.7 (-1.3, 4.7)	0.261
Eating window (h)	-2.0 (-2.6, -1.3)	**<0.001**	-2.2 (-3.5, -0.9)	**0.004**	-1.9 (-2.7, -1.1)	**<0.001**
Caloric midpoint (h)	1.3 (0.5, 2.1)	**0.002**	0.4 (-1.1, 1.8)	0.588	1.7 (0.8, 2.7)	**0.001**
Eating jetlag (h)	0.9 (0.3, 1.5)	**0.003**	1.0 (-0.2, 2.2)	0.095	0.9 (0.2, 1.6)	**0.018**
Time from midsleep point to first food intake (h)	1.8 (1.2, 2.3)	**<0.001**	2.2 (0.9, 3.5)	**0.003**	1.6 (1.0, 2.1)	**<0.001**
Time from last food intake to midsleep point (h)	0.3 (-0.2, 0.8)	0.224	-0.1 (-0.9, 0.8)	0.885	0.5 (-0.2, 1.1)	0.150

Values obtained from Welch's t-test (breakfast skippers – consumers). Cardiometabolic risk score was calculated for each sex based on waist circumference, blood pressure, plasma glucose, high-density lipoprotein cholesterol, and triglyceride concentrations (see methods for further details). A higher MeD-P and MeD-S represents greater adherence to the Mediterranean diet, whereas the higher the MeD-DQI score, the lower the Mediterranean diet quality. A higher DASH score represents greater adherence to the DASH guidelines. A lower DQI score represents a higher diet quality. The higher the DII score, the more inflammatory the diet. Some specific outcomes had missing data for all: [a]2 missing participants (one skipper and one consumer), [b]4 missing participants (two skippers and two consumers), [c]3 missing participants (one skipper and two consumers), [d]7 missing participants (three skippers and four consumers), [e]1 missing subject (one consumer), [f]4 missing participants (one skipper and three consumers), [g]3 missing participants (one skipper and two consumers), [h]2 missing participants (consumers), and [i]4 missing participants (two skippers and two consumers); for men: [a,b,g]2 missing participants (one skipper and one consumer), [c,f,h]1 missing subject (one consumer), [d]3 missing participants (one skipper and two consumers), and [i]3 missing participants (two skippers and one consumer); and for women: [b,c]2 missing participants (one skipper and one consumer), [d]4 missing participants (two skippers and two consumers), [e, g,h,i]1 missing subject (one consumer), and [f]3 missing participants (one skipper and two consumers). Abbreviations: CI, confidence intervals; Cons., breakfast consumers; HOMA-IR, homeostasis model assessment index; MD, mean difference; MeD-P, *a priori* Mediterranean dietary pattern; MeD-S, Mediterranean diet score; MeD-DQI, dietary quality index for the Mediterranean diet; DASH, dietary approaches to stop hypertension; DQI, dietary quality indices; DII, dietary inflammatory index; Skip., breakfast skippers

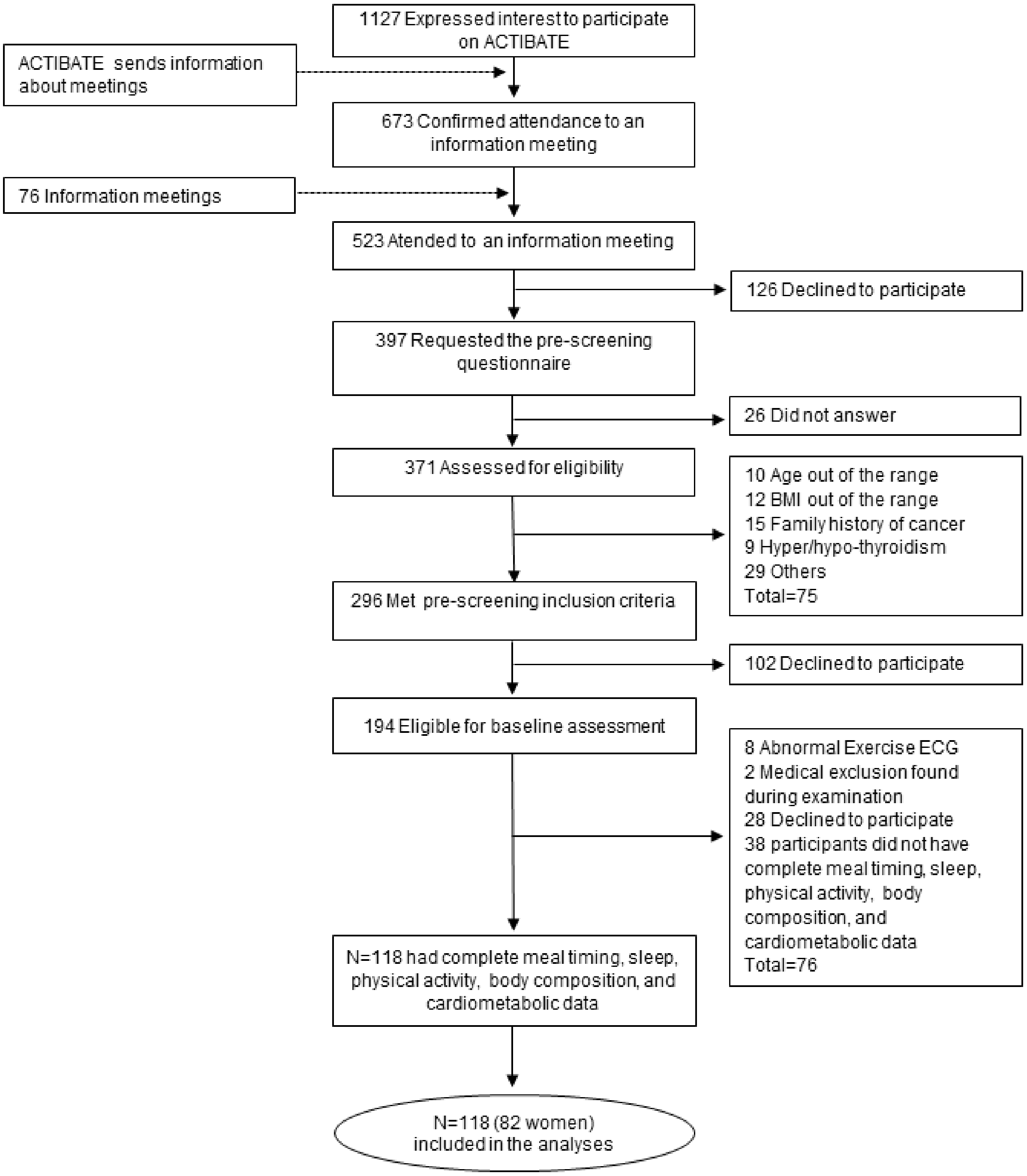

Figure S1. Flow-chart for subject enrolment. BMI: body mass index, ECG: electrocardiogram.

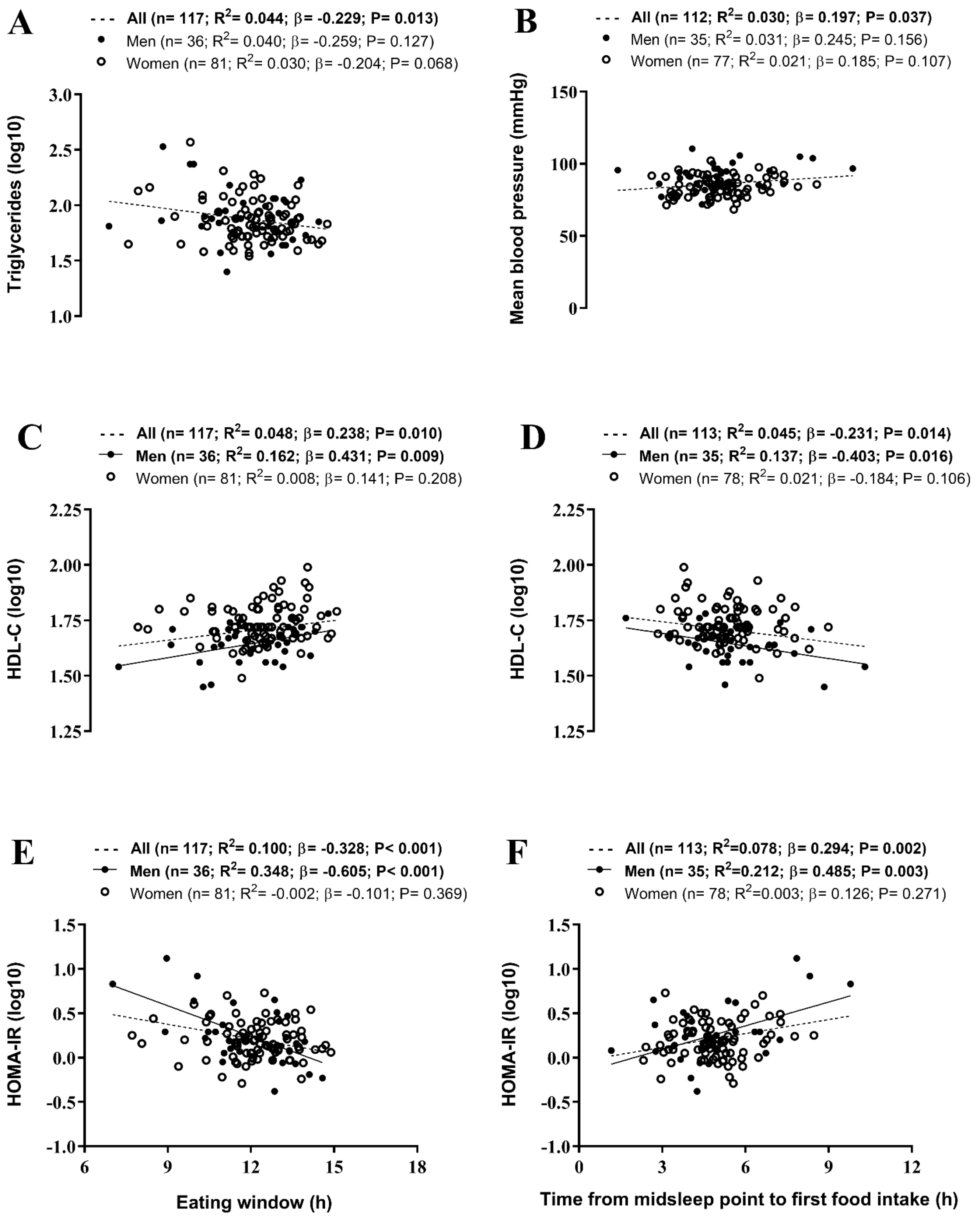

Figure S2. Scatterplots of the associations of eating window and time from midsleep point to first food intake with cardiometabolic risk factors (only significant associations from table 3 are shown) in young adults. Adjusted R^2, β standardized regression coefficients and P values are obtained from single linear regressions. All cardiometabolic risk factors (except for mean blood pressure) were log10-transformed to bring their distributions closer to normal. Abbreviations: HDL-C, high-density lipoprotein cholesterol; HOMA-IR, homeostasis model assessment of insulin resistance.

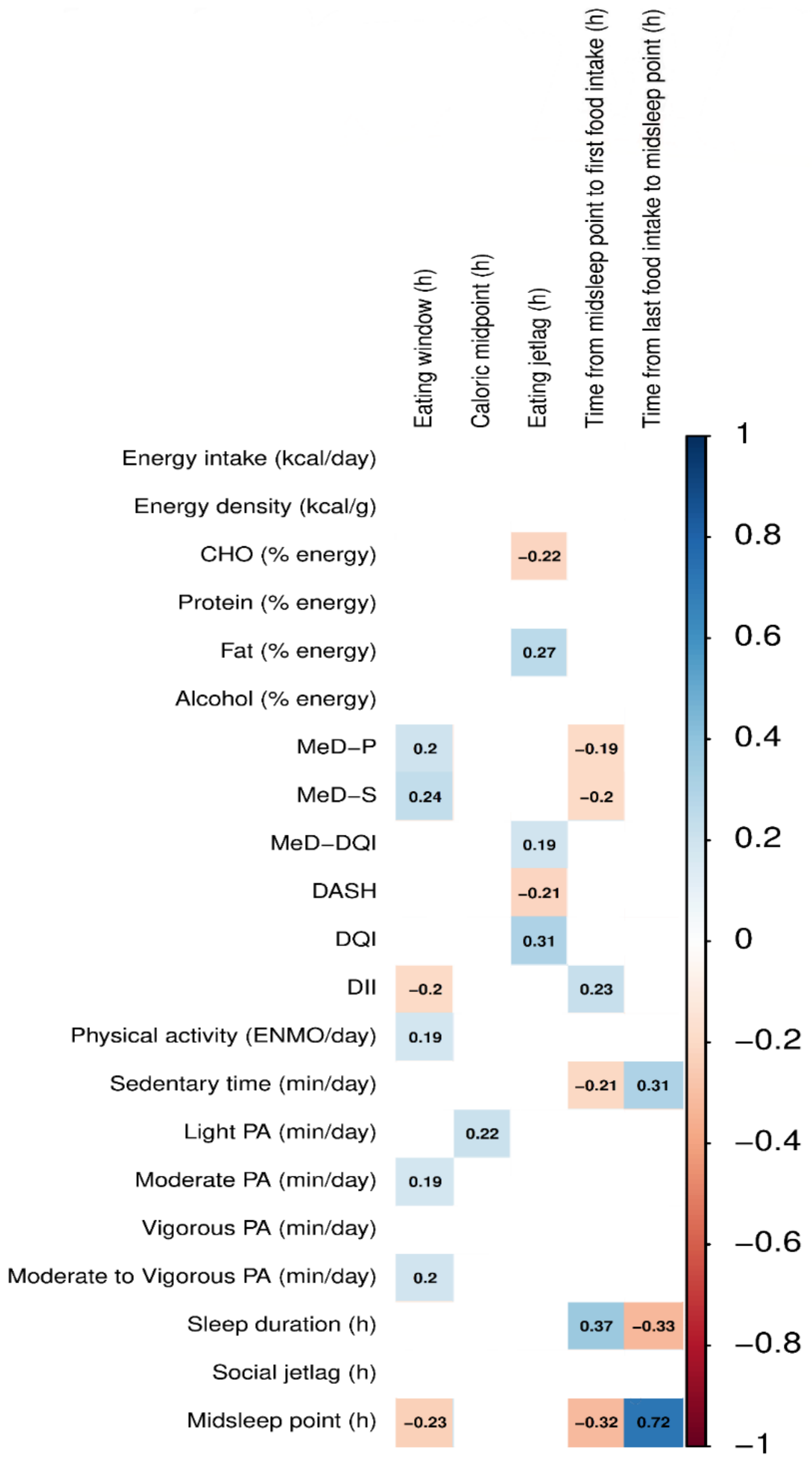

Figure S3. Pearson correlation of meal timing with energy and macronutrients intake, dietary patterns, physical activity and sleep patterns in young adults. Boxes only represent the statistically significant (P≤0.05) correlations and the value within the boxes show the Pearson correlation coefficient. Blue boxes indicate positive correlation whereas red squares indicate negative correlation. Abbreviation: CHO, carbohydrates; MeD-P, *a priori* Mediterranean dietary pattern; MeD-S, Mediterranean diet score; MeD-DQI, dietary quality index for the Mediterranean diet; DASH, dietary approaches to stop hypertension; DQI, dietary quality indices; DII, dietary inflammatory index; PA, physical activity.

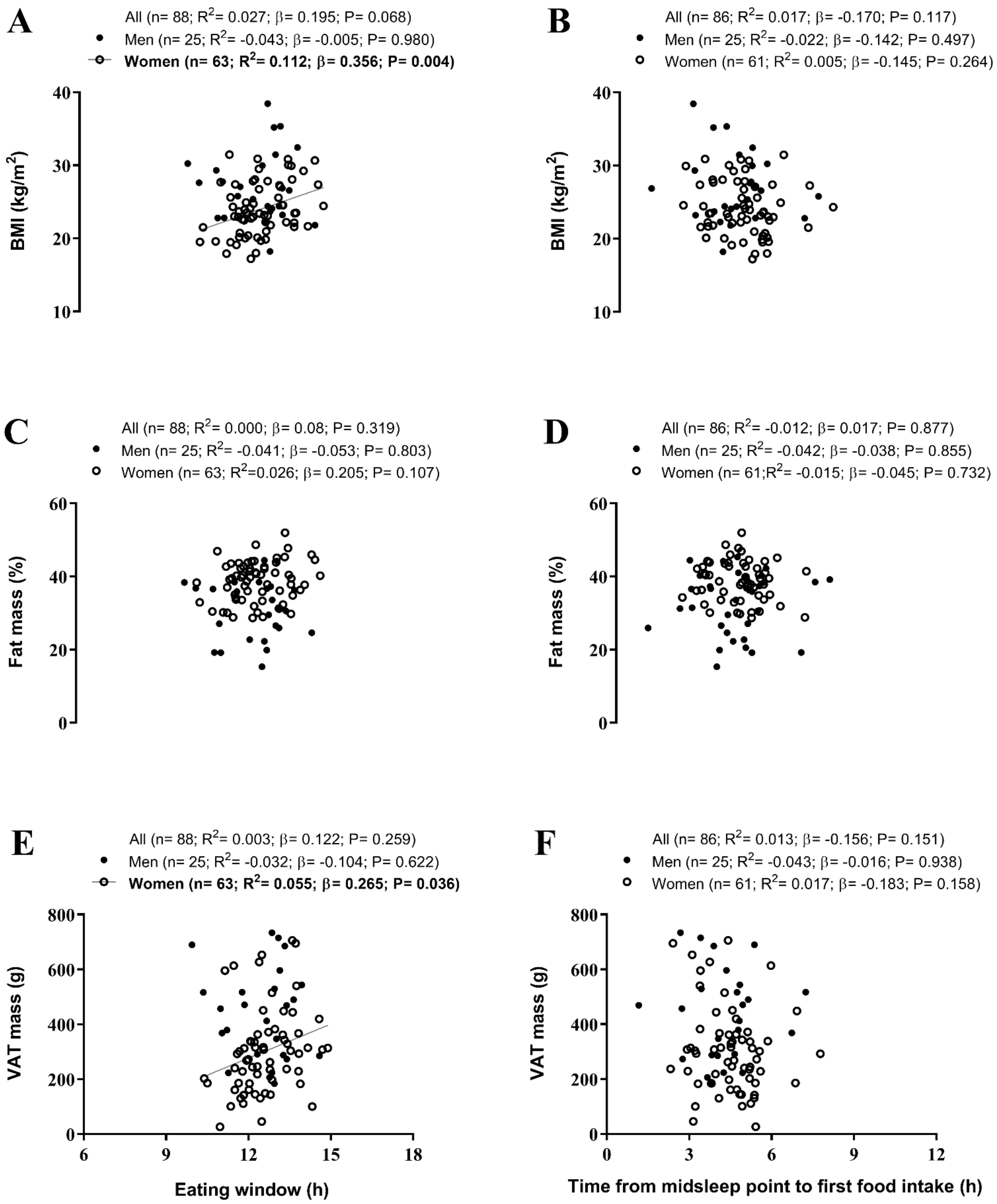

Figure S4. Scatterplots of the associations of eating window and time from midsleep point to first food intake with body composition in breakfast consumers. R^2 adjusted, β standardized regression coefficients and P values are showed from single linear regressions. Abbreviations: BMI, body mass index; VAT, visceral adipose tissue.

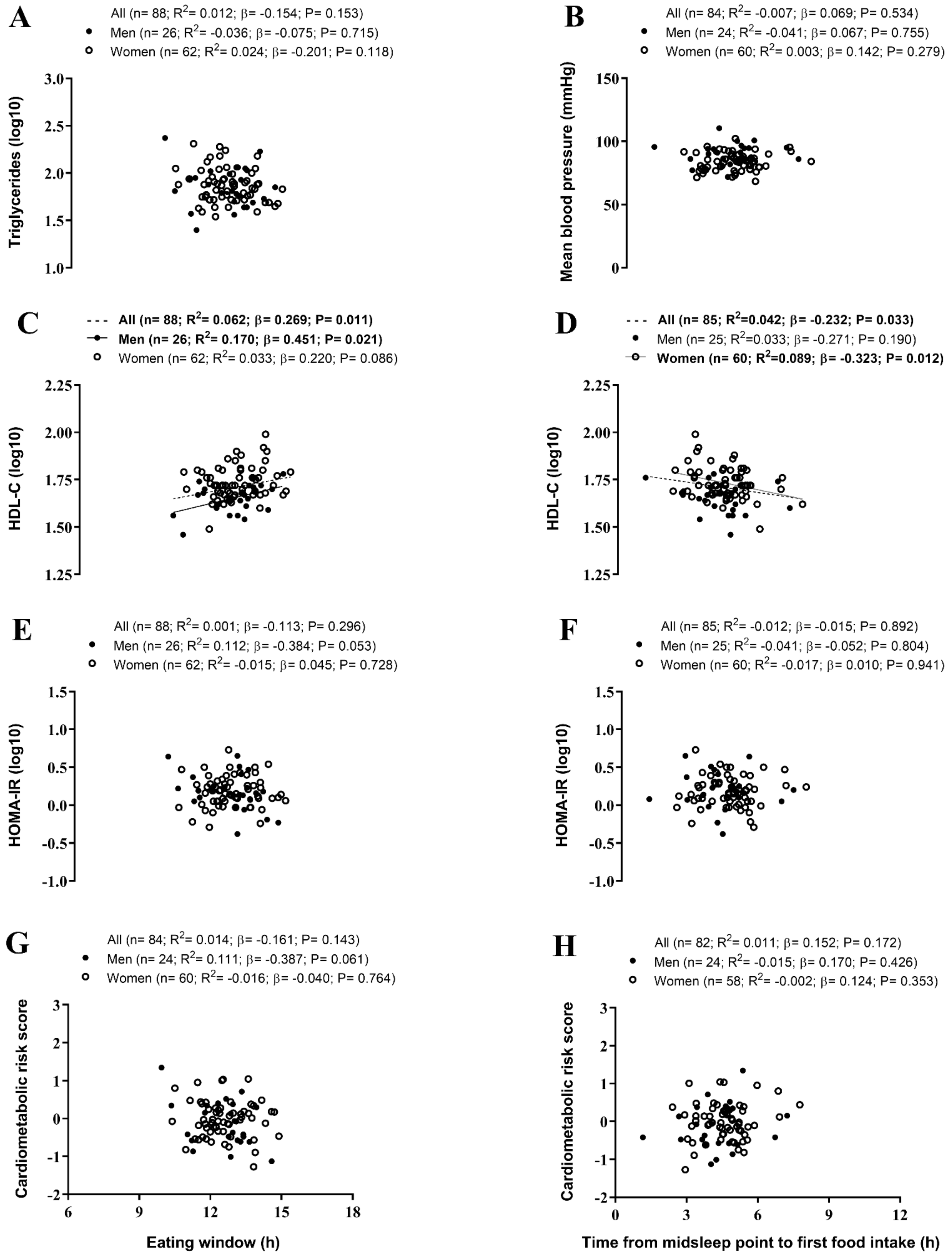

Figure S5. Scatterplots of the associations of eating window and time from midsleep point to first food intake with cardiometabolic risk factors (only those associations which were significant in table 3 are shown) in breakfast consumers. R^2 adjusted, β standardized regression coefficients and P values are showed from single linear regressions. All cardiometabolic risk factors (except for mean blood pressure and cardiometabolic risk score) were log10-transformed to bring their distributions closer to normal. Cardiometabolic risk score was calculated for each sex based on waist circumference, blood pressure, plasma glucose, high-density lipoprotein cholesterol, and triglyceride concentrations (see methods for further details). Abbreviations: HDL-C, high-density lipoprotein cholesterol; HOMA-IR, homeostasis model assessment index.
